# Cow’s Milk Protein Allergy from Diagnosis to Management: A Very Different Journey for General Practitioners and Parents

**DOI:** 10.3390/children2030317

**Published:** 2015-07-21

**Authors:** Adriana C. Lozinsky, Rosan Meyer, Katherine Anagnostou, Robert Dziubak, Kate Reeve, Heather Godwin, Adam T. Fox, Neil Shah

**Affiliations:** 1 Department of Paediatric Gastroenterology, Great Ormond Street Children’s Hospital, London WC1N 3JH, UK; E-Mails: Adriana.LozinskyRolnik@gosh.nhs.uk (A.C.L.); Rosan.Meyer@gosh.nhs.uk (R.M.); Robert.Dziubak@gosh.nhs.uk (R.D.); Kate.Reeve@gosh.nhs.uk (K.R.); Heather.Godwin@gosh.nhs.uk (H.G.); 2 Department of Paediatric Allergy, Guy’s and St Thomas’ Hospitals NHS Foundation Trust, Westminster Bridge Road, London SE1 7EH, UK; E-Mails: aikaterini.anagnostou@gstt.nhs.uk (K.A.); Adam.Fox@gstt.nhs.uk (A.T.F.)

**Keywords:** cow’s milk protein allergy, knowledge, quality of life

## Abstract

Cow’s milk protein allergy (CMPA) is the most common food allergy in infants and can affect a family’s quality of life. The purpose of this paper is to evaluate the knowledge and experience of general practitioners (GPs) in terms of CMPA diagnosis and management and to explore the views of parents on the current diagnostic process. Two surveys were conducted in June 2014, which collected data from GPs and parents of infants diagnosed with CMPA in the United Kingdom. The questionnaires included quantitative and qualitative questions, which measured self-reported knowledge, management and perceived treatment progression, and the educational needs of GPs. We also explored parents’ experiences of local healthcare support in relation to CMPA. A total of 403 GPs and 300 parents completed the surveys. The main symptoms of CMPA and diagnosis period differed between GPs and parents. Other key points include different perceptions on symptom presentation and improvement, lack of awareness from GPs about current guidelines, and the significant burden on both families and GPs. This is the first study attempting to establish GP and parental experience in diagnosing CMPA. It isnotable that the difference can be improved through training, appropriate diagnostic tools and improved communication between physicians and parents.

## 1. Introduction

Cow’s milk protein allergy (CMPA) is the most common food allergy in children, affecting between 1.9%–4.9% of infants [1]. Symptoms tend to develop in the first few months of life and the resolution rates vary according to the type of allergy. Patients with non-immunoglobulin E (IgE)-mediated allergies tend to have outgrown this by 2 years of age [2], while children with IgE-mediated allergies do so at about 3 years old [3].

The guideline from the National Institute of Allergy and Infectious Disease (NIAID) classifies food allergies into three categories: IgE-mediated, non-IgE-mediated and mixed IgE- and non-IgE-mediated [4]. In IgE-mediated CMPA, symptoms can occur immediately and up to 2 h after the ingestion of cow’s milk, and can affect the skin, respiratory system, and gastrointestinal tract and cause severe systemic reactions (anaphylaxis) which are potentially life threatening [4,5]. On the other hand, in non-IgE-mediated CMPA the manifestations of the symptoms can be delayed, up to 72 h after the exposure to cow’s milk depending on the gastrointestinal symptoms involved. The symptoms are not specific and include colicky pain, persistent gastro-oesophageal reflux, diarrhoea or constipation, blood in stools and exacerbation of eczema [4,5]. These can lead to a diagnosis of allergic proctocolitis, food protein-induced enterocolitis syndrome or eosinophilic gastrointestinal diseases, so it is not unusual to have cases of non-IgE-mediated CMPA being misdiagnosed. The most common confusion is with lactose intolerance, which presents with abdominal pain, bloating, flatulence and diarrhoea but usually without atopic co-morbidities [6]. In addition, CMPA manifests in early childhood [4,5], whilst primary lactose intolerance usually occurs from 3 to 4 years of age and secondary lactose intolerance is transient [7]. Infantile colic can also overlap with CMPA and the incorrect recommendation of colic remedies and anti-colic formulas can delay the correct diagnosis [8].

The first step for the diagnosis is an allergy-focused history which includes the assessment of symptoms, the identification of the different underlying immunological mechanisms, and then linking this to suspected foods [9,10]. For IgE-mediated CMPA, skin prick tests or specific IgE blood tests are useful tools in the diagnostic process. In suspected non-IgE-mediated CMPA, the allergy tests have limited value. The oral food challenge test remains the gold standard for both types of allergy. In some cases, however, it is not performed when there is significant clinical history and laboratory data [11]. The treatment of CMPA is the complete removal of cow’s milk from the child’s diet, which may in some cases also be required for the breast-feeding mother. Ideally this should be guided by a qualified dietitian [5,12].

In recent years, guidelines on the diagnosis and management of CMPA have been published, with sources including the European Society of Paediatric Gastroenterology, Hepatology and Nutrition (ESPGHAN) [12], the European Academy of Allergy and Clinical Immunology (EAACI) [9], the Diagnosis and Rationale for Action against Cow’s Milk Allergy (DRACMA) [1]. Three guidelines exist in the United Kingdom (UK) which include the National Institute for Clinical Excellence Guidelines (NICE) [5] on food allergy diagnosis and two published guidelines specific for CMPA: one aimed at primary care (Milk Allergy in Primary Care (MAP) guidelines) [13] and the other for secondary and tertiary care, published by the British Society for Allergy and Immunology [14]. Both cover common presenting symptoms and suggest tests and treatment options that should be sufficient to diagnose and optimally manage children with this allergy.

In spite of these guideline documents, significant delays in diagnosis and optimal management continue [15]. This has also been our experience with many parents reporting poor recognition at initial presentation and suboptimal nutritional management. The aim of this survey was therefore to evaluate the experience of CMPA that general practitioners (GPs) have, as the first point of contact for children with suspected allergies in the UK, and to explore the views of parents on the current diagnostic process for CMPA, to enable better management in the future.

## 2. Methods

Two separate surveys were conducted in June 2014, one for GPs and one for parents. The survey for the GPs was developed by Mead Johnson Nutrition UK and medeConnect (http://www.medeconnect.net/), a leading provider of community-based healthcare research in the UK using both online quantitative and qualitative research methods. The parent questionnaire was developed by Mead Johnson Nutrition UK and Opinion Health (http://opinionhealth.com/about_us), a company that specialises in providing patient insight into healthcare. Both questionnaires were reviewed by Allergy UK (www.Allergy.uk), a leading charity supporting healthcare professionals and patients with food allergies.

GPs who had seen at least one case of a child with CMPA in the past year and parents of an infant diagnosed with CMPA in the past year were eligible to take part in the survey. Ethical approval was not required from the UK ethics authorities as it fell into the category of an anonymous online survey.

The GP survey was distributed online via www.Doctors.net.uk. This site is the largest and most active network of General Medical Council-authenticated doctors in the UK. It is a trusted channel for information, communication and education and is used by more than 40,000 doctors every day. Currently it is the leading channel for communication and research for doctors in the UK. The GPs’ survey recorded demographics of the physicians and measured self-reported knowledge on CMPA diagnosis, management and educational needs, and barriers to the diagnosis of CMPA. The questionnaire had 35 questions with both open and closed (categorised) questions.

The second online survey for parents explored similar topics, including the burden to the family, but also contained qualitative data on management experience with CMPA. The survey was sent to everybody that was registered on the database of Opinion Health (www.Opinionhealth.com). The questionnaire was developed to better understand of the role and attitudes of parents in the diagnostic process for CMPA.

### Statistical Analysis

Statistical analysis was performed using IBM SPSS Statistics for Windows, version 21 (Armonk, NY, USA). Descriptive statistics are presented in percentage and figures.

## 3. Results

### 3.1. Characteristics of GPs and Parents

**Figure 1:**
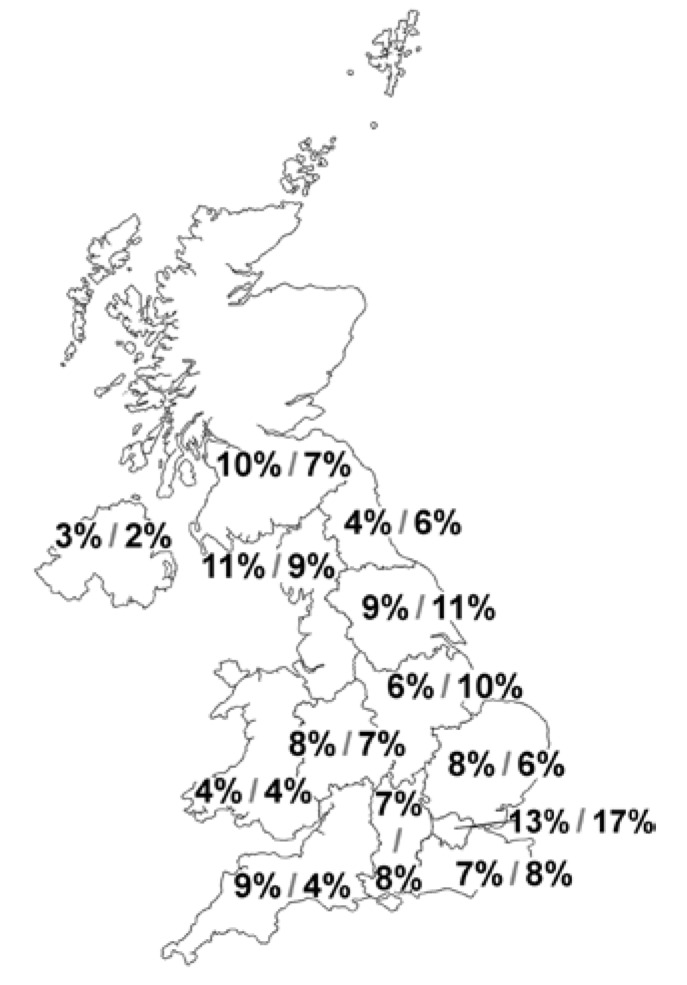
Geographical distribution of participating GPs’ practice and parents (%GP/%Parent).

A total of 403 GPs completed the survey, and their distribution was from all areas of the UK (Figure 1). Fifty-five per cent were male, 75% graduated between 1990 and 2011 and the majority (67%) of them were experienced GPs. Sixty-two per cent of the GPs (251/403) worked full time and 14% of them had a dietitian associated with their practice. In the 12-month period prior to completing the survey, 52% of the GPs diagnosed ≥ 3 children with CMPA and 65% managed ≥3 cases already diagnosed with this allergy (Table 1).

**Table 1:** Summary of GPs’ practice.

Number of Infants <1 Year Diagnosed as CMPA (12 Month Period)	*n*	%
1–2	192	48%
3–4	86	21%
5–10	94	23%
11+	31	8%
**Number of Infants <1 Year Treated for CMPA (12 Month Period)**	***n***	**%**
None	40	10%
1–2	98	24%
3–4	102	25%
5–10	113	28%
11+	50	12%

A total of 300 parents of infants diagnosed with CMPA completed the second survey questionnaire, of which 206 (68.7%) were mothers and 94 were fathers. Twenty per cent of the parents were aged between 18 and 24 years, with the majority (57%) between 25 and 34 years, and 24% were 35 years old and older. Twenty eight per cent had 1 child, 40% had 2 children and 31% 3 or more children. The parents’ distribution was from all areas of the UK (Figure 1).

### 3.2. Symptom Presentation to Diagnosis: Parents vs. GPs

**Figure 2:**
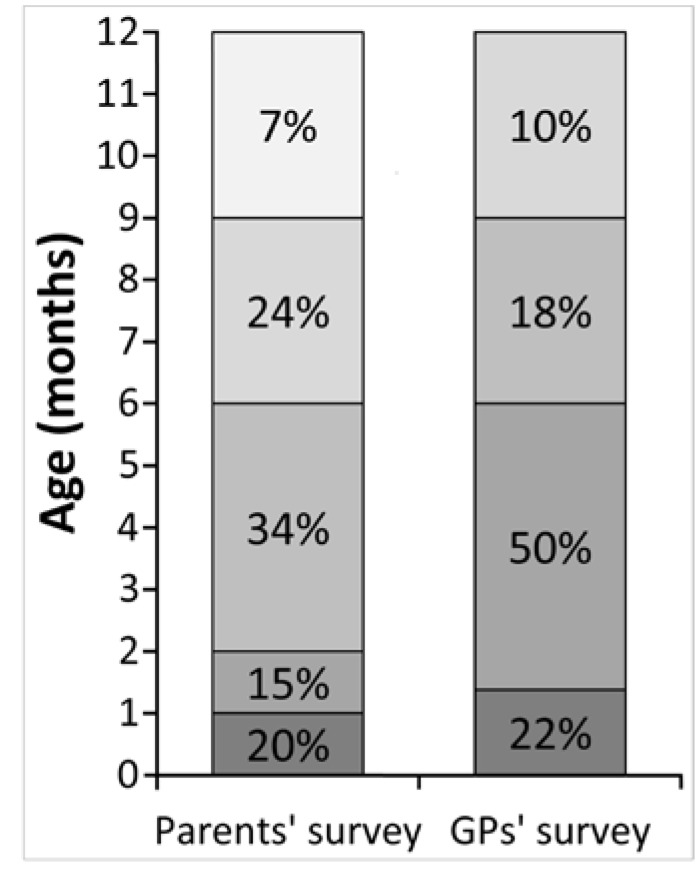
How old the child was during the first appointment with the GP.

Parents reported that 49% per cent of the patients presented with symptoms within the first three months of life, and the most common symptoms were colicky pain (60.7%), nausea and vomiting (52%), diarrhoea (51%) and eczema (36%). Similarly, the GPs reported that 72% of children with a suspected CMPA presented to their practice before 6 months of age and their most common symptoms of CMPA included diarrhoea (59%), nausea and vomiting (40%), eczema (31%) and colicky pain (24%) (Figure 2).

In 49% of cases, parents reported that the diagnosis of their child’s CMPA occurred within the first 6 months of life and it took on average 10 weeks and a median of four visits to a health care professional before a diagnosis of CMPA was made. In 48% of cases, the diagnosis was made by the GP followed by 21% by paediatricians, 13% by the health visitor and in 5% by a dietitian. Prior to seeking advice from health care professionals, 46% of the parents tried to commence a self-directed elimination diet, 8% used high street tests to “aid” diagnosis and 7% used homeopathy as part of management.

On the other hand, the GP survey indicated that it took a median of 8 weeks to diagnose CMPA but acknowledged that it ideally should take 4 weeks based on current guidelines. When GPs were asked to distinguish between IgE and non-IgE symptoms, 4% of GPs listed anaphylaxis as a non-IgE CMPA symptom. Seventy per cent responded that it was “not necessary to perform any tests” for the diagnosis of CMPA, 17% used specific IgE blood tests and 12% used skin prick tests. When CMPA was suspected, other common conditions considered by the GPs were colic (88%), lactose intolerance (79%), gastro-oesophageal reflux disease (64%) and atopic eczema (78%). The most common medications prescribed by GPs were: emollients (76%), colic relief (*i.e.*, Simeticone) (61%), steroid cream (41%) and antihistamines (27%). Adrenaline auto-injectors were prescribed in 2% of the CMPA children.

Fifty one per cent of GPs consulted the CMPA guidelines, though it was not clear which version was being used, to guide their management, and the same percentage reported going ahead with removing CMP from the infant’s diet and prescribing a hypoallergenic formula when this allergy was suspected. However, 45% of infants with this suspected diagnosis were referred to secondary care as part of the diagnostic process.

### 3.3. Burden of Disease on GPs and Families

Eighty-seven per cent of the GPs found themselves over-stretched in terms of time, resources and costs and 39% said that in the national healthcare system, “the pressure to meet budgets had a negative impact on their ability to treat patients” with CMPA. More than half of them agreed that “the delayed diagnosis of CMPA adds even more pressure to a (national) primary care system already struggling with ever increasing demands”, with more than half (53%) stating that they “feel more confident involving secondary care in the diagnosis”. Better and faster access to various secondary care specialists is seen as a way of speeding up diagnosis for 31% of the GPs.

The GPs thought that the biggest communication challenges in relation to the parents of children with CMPA were: excessive worry or stress associated with their child’s CMPA (79%), unrealistic expectations about their condition and management (61%) and the continued parental request for specific prescriptions of hypoallergenic formulas (60%).

From the parent survey, the significant burden on both parent and child was evident, in particular the length of time to reach a diagnosis which has a negative impact on the child, along with poor sleeping (45%), persistent crying (37%) or abdominal pain (41%). The documented adverse impact on the parents’ life included the feeling of exhaustion in 46.7% which was mainly related to lack of sleep, 55.7% had stress or anxiety related to their child’s health and 33% of fathers reported a delay in going back to work due to their child’s health problems and the time taken to reach a diagnosis of CMPA. The parents mentioned a negative impact on their family life too, with 36.2% stating that they could not enjoy time together as a family and 28% thought their other children got less time and attention.

Parents were also made to feel like they were overreacting, worrying too much about nothing and were not taken seriously in 56% of cases. Forty-six per cent of the parents felt that the amount of time taken to reach the diagnosis of CMPA had impacted the relationship between parents and child, with 16% of fathers and 23.2% of mothers reporting often feeling very irritated and frustrated with the baby and 21% (11.7% of fathers and 25.2% of mothers) felt very low or depressed. Of these 63 parents, 39.7% sought support from a health care professional for feelings of depression.

### 3.4. Perception of Knowledge/Training by GPs and Parents

The majority of GPs according to this survey were not familiar with the current guidelines for CMPA. Only 13% consider themselves “very familiar” with the NICE allergy guidelines and 6% are following it. Five per cent of GPs reported to be “very familiar” with UK CMPA guidelines and 2% with ESPGHAN guidelines (Table 2).

In the last five years, 69% of the GPs received 1 to 5 h of training in paediatric allergy and 46% rated the training as not effective. The vast majority (82%) of GPs felt they would benefit from further education and support on CMPA. In addition, it was also reported that in 31% of cases, faster access to secondary care for diagnosis or advice would improve the diagnosis. The GPs also highlighted that parents could help speed up the diagnosis of CMPA by filling in a food/symptoms questionnaire (29%), prompting the GP during consultation and being persistent (19%) as well as attending regular appointments and follow-ups (17%).

**Table 2:** Knowledge of current guidelines on CMPA.

Guideline	Not Aware	Aware but not Read	Somewhat Familiar	Very Familiar/Not Following	Very Familiar/Following
NICE	18%	24%	45%	6%	7%
MAP	55%	23%	17%	3%	2%
ESPGHAN	81%	13%	4%	1%	0%

On the other hand 46% of the parents stated that their GP did not seem to know a lot about CMPA. The survey captured their response and advice to other parents with the following quotes:

- “Go see a doctor, use your instincts and don’t doubt yourself!”
- “Push push push until someone takes notice”
- “Keep going back to see professionals and insist on referral to a paediatrician and dietitian.”
- “Don’t take no for an answer and keep chasing appointments. Do not give up.”

## 4. Discussion

**Figure 3:**
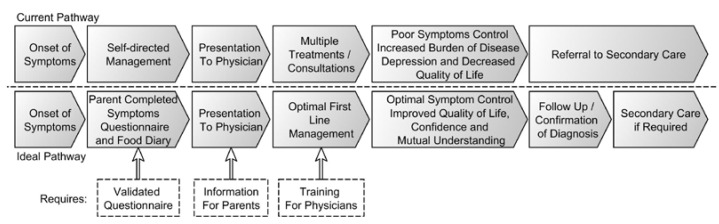
Discord in journey.

To the best knowledge of the authors, this is the first publication that reports on the journey from diagnosis to management in CMPA as experienced by GPs and parents who have a child with this diagnosis. This study evaluates self-reported knowledge on CMPA, the diagnosis, and treatment and educational needs among GPs in the UK, and it also aimed to establish parents’ perceptions and experiences with their own child with CMPA and the burden of the disease on the families. From these results, it is clear that there are significant differences in perceptions between the GP and the parent of the child with CMPA, which is to be expected. In many areas, however, the opposing views highlight shortcomings in medical management and improvements in communication that could ultimately improve the management of CMPA (Figure 3).

The age of presentation of the first symptoms and the visit to their GP correlated between parents and GPs. This confirms the current research that the majority of infants with CMPA will present within the first 6 months of life [16]. Although there is significant overlap in symptom reporting, it is interesting that colicky pain is listed as the most important presenting symptom by parents but not by the GPs. Hill *et al.* [8] highlighted for the first time in 1995 the overlapping disease profile between IgE and non-IgE-mediated allergies, with reflux and colic as part of this overlapping disease. Since then several studies have found that in some children, colic may be related to CMPA [17,18]. In the study by Meyer *et al.* [19], 90% of children that presented with a non-IgE-mediated allergy affecting their gastrointestinal tract had abdominal pain. It is therefore a feature that is commonly described but the severity is difficult to assess for physicians, as it is so subjective. We hypothesise that this survey has highlighted this as a main presenting symptom for parents because colicky pain affects sleep for both child and parents, which can impact family life and the relationship between parent and child.

According to the survey on parents, it took on average of 10 weeks for the diagnosis to be made, whereas GPs estimated this to occur within 8weeks. It is however acknowledged that it should ideally occur within 4 weeks’ time. This finding is supported by a study from Sladcevicius *et al.* who showed that it took on average 9 weeks with numerous GP visits in the UK before a child was diagnosed correctly with CMPA [15]. Conversely, a similar study in the Netherlands found that the diagnosis of CMPA occurred there within 4 weeks [20]. This is an important finding that needs to be addressed, as Valentine *et al.* found that the combinations of the symptoms of CMPA with the delayed diagnosis in some cases may affect a family’s dynamic and quality of life [21]. In addition there are some studies on IgE-mediated food allergies that showed a significant impact on the parents’ life, possibly because of the combination of the child’s symptoms, extra-intestinal manifestations (for example poor sleep) [22], the difficulty of getting a quick diagnosis and correct treatment, and the burden and difficulty of following an elimination diet [23]. A delay in diagnosis may also lead to feeding difficulties due to on-going pain associated with feeding, leading to a negative association with the breast or bottle, and may also disrupt early weaning [24,25]. In 2011, Saps *et al.* found that CMPA in early infancy increases the risk of developing functional gastrointestinal disorders in later childhood, such as abdominal pain [26].

The lack of awareness of guidelines on CMPA and the training identified as necessary on this topic has bearing on the recognition and management of this food allergy (Table 3). Our data is supported by a larger study by Hazeldine *et al.* [27] on GPs’ perceptions of UK allergy care. More than 50% rated allergy care in primary care as poor and 40% reported that they would benefit from more training. This lack of knowledge was also identified as a barrier for parents (Table 3). Although it may be perceived as a problem unique to the UK as GPs are the first point of contact for the recognition of CMPA, data implies that there are similar knowledge barriers for paediatricians worldwide. Quitadamo *et al.* published in 2014 a result from a survey that evaluated the knowledge about gastro-oesophageal reflux guidelines and found that the majority of the paediatricians were unaware of the 2009 guideline from ESPGHAN and the North American Society for Pediatric, Gastroenterology, Hepatology and Nutrition [27]. In 2014, Maslin *et al.* [28] published a survey on food allergy competencies amongst UK, Australian and USA dietitians, and found a similar trend in physicians with a need to increase their knowledge in different aspects of food allergy diagnosis and management, specifically the areas of developing food challenge protocols and management of feeding problems. As a result, the UK has implemented competency-based training courses on CMPA for dietitians [28]. We believe that such a training program for physicians would benefit and improve diagnosis and management. Whilst awaiting the development of these formal training programs, tools to guide diagnosis may also be useful. A recent tool developed to help GPs with cases of suspected CMPA and/or to evaluate improvement of symptoms is the Cow’s Milk-related Symptom Score (CoMiss), published by Vandeplas *et al.* which comprises general symptoms, gastrointestinal, dermatological and respiratory manifestations common to children with CMPA. This tool has not been validated yet but may prove to be useful in primary care [29].

A very important finding of this survey is the impact of the response from the GPs on reported symptoms from parents and on-going management. On the one hand, the GPs found parents to have excessive worry or stress in relation to their child’s allergy with unrealistic expectations, but on the other hand, parents did not think they were taken seriously and that they were worrying unnecessarily. This discord may lead to increased worry and stress and may also exacerbate the request for further formula changes as perceived by the GP (Table 3). Many of our parents reported feeling depressed about their child’s diagnosis, which has been shown to have impact on communication and also satisfaction with the service. In 2012, Fagnano *et al.* [30] studied parents of children with asthma and the impact of depression on the communication between parents and health care professionals. In that study, 30% of parents had depressive symptoms, and that group was less satisfied with professional visits and felt that their needs were not met.

This study has many limitations. Firstly, a response bias exists, mainly from parents, as those who had a bad experience with their children’s diagnosis of CMPA are more likely to participate in such a survey. The second limitation is that it was an online survey distributed via social networks and the questions were self-rated, so some results could be subjective. It is important to highlight that, in the GPs’ survey, the responses were more focused on non-IgE conditions and just few a questions targeted IgE-mediated allergies. This was because health economic studies (e.g., Sladkevicius *et al.*) [15] demonstrate that the diagnostic delay is most common in these patients.

**Table 3:** Summary of suggestions from this survey to improve the diagnosis of CMPA.

Suggested Actions for GPs	Suggested Actions for Parents
Improve listening skills	Develop a symptoms diary that can be taken to the appointment
Allow more time for an allergy-focused history	Develop a simple food diary that can be taken to the appointment
More training on how to recognise and manage	Create awareness that there are guidelines for recognition and management of CMPA
Improve awareness of current guidelines	Create more awareness of CMPA through parent teaching/online training
Development of tools to aid diagnosis and management	Create leaflets with explanation of symptoms/diagnosis/treatment
More access to secondary and tertiary care	

## 5. Conclusions

This is the first study attempting to establish the experience of both GPs’ and the parents’ journey from diagnosis to management of CMPA. The difference between parents’ and GPs’ views on presentation and management of CMPA is notable, which has clinical implications for not only the child but also the family. From this survey, it is clear that GPs require more education on CMPA and also need tools to make this task easier for them. Parents, on the other hand, can also help with this process by improving their knowledge about the condition and making use of tools that they can complete prior to attending an appointment. There is a lack of validated tools to assist with the diagnosis of CMPA, and this survey highlights the need for these to be developed for primary care.

## Abbreviations

CMPA	Cow’s milk protein allergy
GP	general practitioner
UK	United Kingdom
NIAID	National Institute of Allergy and Infectious Disease
IgE	Immunoglobulin E
ESPGHAN	European Society of Paediatric Gastroenterology, Hepatology and Nutrition
EAACI	European Academy of Allergy and Clinical Immunology
DRACMA	Diagnosis and Rationale for Action against Cow’s Milk Allergy
NICE	National Institute for Clinical Excellence Guidelines
MAP	on food allergy diagnosis, Milk Allergy in Primary Care
